# Supplementary material for: Biochemical and Structural Characterization of a Highly Glucose-Tolerant β-Glucosidase from the Termite Reticulitermes perilucifugus
Source: Int J Mol Sci. 2025 Mar 28;26(7):3118. doi: 10.3390/ijms26073118 (PMC11989107; doi:10.3390/ijms26073118)
Supplement: Supplementary file 1 [file ijms-26-03118-s001.zip › ijms-3550050-supplementary.pdf]

Supplementary Files for Article

Biochemical and Structural Characterization of a Highly Glucose-Tolerant β-Glucosidase from the Termite *Reticulitermes perilucifugus*

Guotao Mao [1,2], Ming Song [1], Hao Li [1], Junhan Lin [1], Kai Wang [1], Qian Liu [1], Zengping Su [1,2], Hongsen Zhang [1,2], Lijuan Su [1], Hui Xie [1,2,*] and Andong Song [1,2,*]

[1] College of Life Sciences, Henan Agricultural University, Zhengzhou 450046, China; maoguotao@henau.edu.cn (G.M.); songming@stu.henau.edu.cn (M.S.); 15993669760@163.com (H.L.); linjunhan@stu.henau.edu.cn (J.L.); kaiwang@gs.zzu.edu.cn (K.W.); 18703662970@163.com (Q.L.); suzengping@henau.edu.cn (Z.S.); hszhang@henau.edu.cn (H.Z.); sulijuan@henau.edu.cn (L.S.)

[2] The Key Laboratory of Enzyme Engineering of Agricultural Microbiology, Ministry of Agriculture and Rural Affairs, Henan Agricultural University, Zhengzhou 450046, China

* Correspondence: xiehui@henau.edu.cn (H.X.); songandong@henau.edu.cn (A.S.)

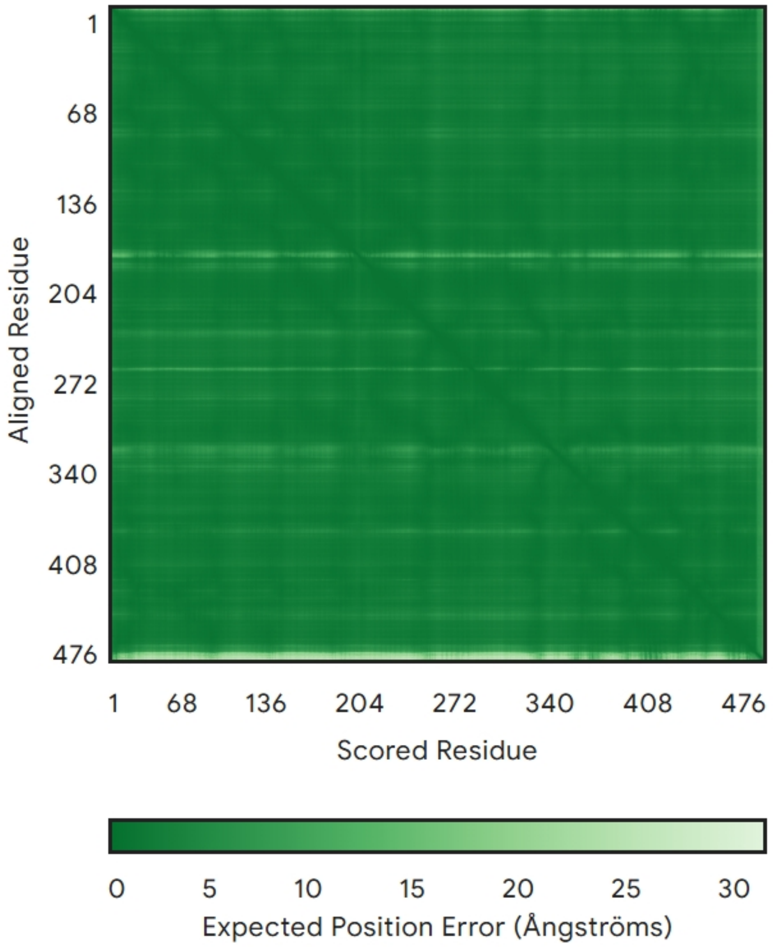

Figure S1. The quality of the structure model of RpBgl8.

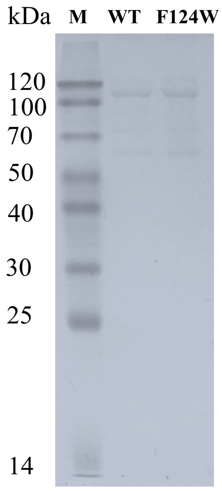

Figure S2. SDS-PAGE analysis of RpBgl8 purified using Ni-NTA chromatography. Lane M: protein marker; WT: the purified wild type RpBgl8; F124W: the purified RpBgl8 viriant F124W.

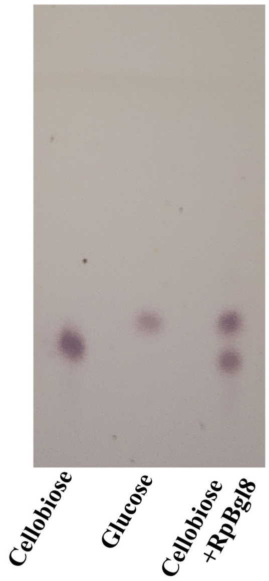

Figure S3. The hydrolysis of cellobiose catalyzed by RpBgl8. After incubation at 37 °C for 1 h in phosphate buffer pH 7.0 containing 20 mM cellobiose, the hydrolysis of cellobiose was determined by thin-layer chromatography.
